# Psychophysiology of Refractive Accommodative Esotropia

**DOI:** 10.1155/2014/927839

**Published:** 2014-11-10

**Authors:** Ungsoo Samuel Kim

**Affiliations:** ^1^Department of Ophthalmology, Kim's Eye Hospital, Konyang University College of Medicine, Youngdeungpo 4th 156, Youngdeungpo-gu, Seoul 150-034, Republic of Korea; ^2^Department of Ophthalmology, Konyang University College of Medicine, Daejeon 302-718, Republic of Korea

## Abstract

*Purpose*. To investigate the psychophysiologic aspects of refractive accommodative esotropia (RAE). *Methods*. I prospectively recruited patients aged 3–6 years with more than 3.0 diopters of hyperopia who presented at Kim's Eye Hospital from January 2011 to March 2013. I compared Korean Child Behavior Checklist (K-CBCL) which consists of internalizing factors (social withdrawal, somatic complaints, anxiety, and depression) and externalizing factors (social problems, thought problems, attention problems, delinquent behavior, and aggressive behavior) between RAE group and control group. *Results*. Two out of three internalizing indexes were significantly different between groups (somatic complaints: RAE children 50.1 ± 4.6 and controls 46.6 ± 5.8, *P* = 0.026; depression/anxiety: RAE children 48.8 ± 7.9 and controls 43.9 ± 6.8, *P* = 0.024). Although there was no significant difference, RAE children scored slightly higher on the externalizing behavior index. In the RAE group, the far angle of esodeviation showed a moderate correlation with withdrawn behaviors. *Conclusion*. Hypermetropic children with high scores on the somatic complaint and depression/anxiety subscales of the CBCL could be at high risk for developing RAE. Psychosocial problems might be related to the pathogenesis of refractive accommodative esotropia.

## 1. Introduction

Refractive accommodative esotropia (RAE) is an esotropia that is restored to orthotropia at all fixation distances and all gaze positions by optical correction of the underlying hypermetropic refractive error [1]. Two main mechanisms contribute to RAE: uncorrected hyperopia and insufficient fusional divergence [2]. Retinal image blur induces accommodation to focus the image; then, this accommodative effort elicits accommodative convergence when fixating at distance or near. When fusional divergence is insufficient to compensate for the excessive convergence innervation, esotropia develops. Vergence movements including divergence require the cooperation of the cerebral cortex, in particular, a state of visual attention called the psychooptical reflex [3].

The Child Behavior Checklist (CBCL) is widely used in the field of psychology and consists of 113 questions used to rate the behavioral and emotional problems of children [4]. There are two dimensions: internalizing symptoms (withdrawn, somatic complaints, and depression/anxiety) and externalizing symptoms (social problems, thought problems, attention problems, delinquent behavior, and aggressive behavior). Therefore, I evaluated the relationships between RAE and psychological symptoms as measured by the CBCL.

## 2. Methods

I prospectively recruited patients aged 3–6 years with more than 3.0 diopters of hyperopia who presented at Kim's Eye Hospital from January 2011 to March 2013. This research study was reviewed and approved by the Institutional Review Board (IRB) of Kim's Eye Hospital, and all procedures conformed to the guidelines of the Declaration of Helsinki.

All patients underwent a full ophthalmologic examination including slit-lamp biomicroscopy, fundoscopy, alternate prism cover testing, and cycloplegic refraction using 1% cyclopentolate. Refractive errors were defined as the spherical equivalent. Refractive accommodative esotropia was defined as an esotropia that was corrected to >10 prism diopters (PD) of orthotropia at both far and near distance with use of the full cycloplegic hyperopic correction. The control group had hyperopia of >3 diopters (D) and orthotropia at near and far distance. Subjects were excluded if they had worn glasses previously or had a history of ophthalmic surgery. Further, children with anisometropia >1.0 D and astigmatism >1.5 D were excluded. The angle of esodeviation was measured by alternate cover test, and the mean deviation of two consecutive measurements was used.

### 2.1. Korean Child Behavior Checklist (K-CBCL)

The K-CBCL was completed by parents at the first visit before glasses were prescribed. The Child Behavior Checklist (CBCL) is a parent-report questionnaire in which the child is rated on various behavioral and emotional behaviors. The reliability and validity of the Korean version of the CBCL (K-CBCL) are well established in Korean children and adolescents [5]. The checklist assesses internalizing (i.e., anxious, depressive, and overcontrolled) and externalizing (i.e., aggressive, hyperactive, noncompliant, and undercontrolled) behaviors. Several subareas are measured, including internalizing factors (social withdrawal, somatic complaints, anxiety, and depression) and externalizing factors (social problems, thought problems, attention problems, delinquent behavior, and aggressive behavior). The K-CBCL score was computed based on Korean normative samples, with the total problem behavior score computed by summing the scores obtained for each item. Raw scores for each clinical factor were transformed into T-scores based on published norms.

The primary outcome was the difference in K-CBCL scores between groups. I investigated the relationship between the K-CBCL score and the amount of esodeviation in RAE children. The data were analyzed using SPSS version 15.0 (SPSS Inc., Chicago, IL). Independent Student's* t*-test was performed to investigate the differences in K-CBCL indexes between groups. The correlation between K-CBCL score and the angle of esodeviation was measured using the Pearson correlation method. Differences were considered significant at *P* values < 0.05.

## 3. Results 

A total of 20 RAE children and 34 hyperopic children were included in the study. The mean ages of RAE children and controls were 5.0 ± 1.7 and 5.4 ± 0.8, respectively (*P* = 0.233). Mean refractive errors were slightly higher in the control group (RAE children, 4.6 D; control group, 5.3 D); however, there was no significant difference between groups (Table 1).

**Figure 1:**
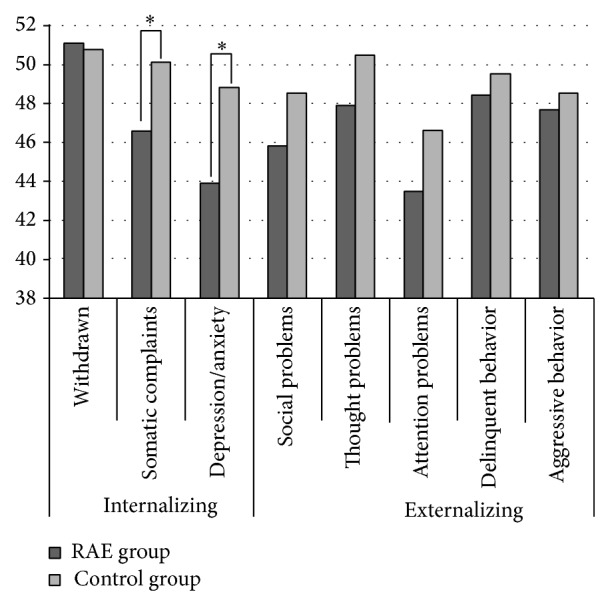
The results of indexes of K-CBCL.

Figure 1 compares K-CBCL in RAE children versus controls. Two out of three internalizing indexes were significantly different between groups (somatic complaints: RAE children 50.1 ± 4.6 and controls   46.6 ± 5.8, *P* = 0.026; depression/anxiety: RAE children 48.8 ± 7.9 and controls 43.9 ± 6.8, *P* = 0.024). Although there was no significant difference, RAE children scored slightly higher on the externalizing behavior index.

Regarding the relationship between the angle of esodeviation and the K-CBCL score, although the frequency of withdrawn behavior did not differ between groups, the far angle of esodeviation showed a moderate correlation with withdrawn behaviors (Table 2). Among the RAE children who scored higher on indices related to social and attention problem, the near angle of esodeviation was larger than the far angle of esodeviation (*r* = 0.725 and *r* = 0.650; *P* = 0.018 and *P* = 0.042, resp.).

## 4. Discussion

The present study revealed that psychosocial problems might be related to the pathogenesis of refractive accommodative esotropia.

Hypermetropic children with several characteristics including a positive family history, low binocular sensory function, low hypermetropia (< +3.0 D), and significant anisometropia had a high risk of developing refractive accommodative esotropia [6]. Unfortunately, I did not investigate sensory function and family history in the present study. Mean refractive error was slightly reduced in the RAE group (RAE children 4.6 D and control group 5.3 D), though this trend was not significant.

The present study showed that RAE children scored higher than controls on the indexes for somatic complaints and depression/anxiety. Somatic complaints include fatigue, aches, nausea, vomiting, headaches, dizziness, and skin, stomach, or eye problems. Children with significant medical disorders tend to score high on the internalizing subscale and particularly on the somatic complaints subscale of the CBCL. Somatic complaints are a predominant characteristic of anxiety disorders and are believed to play an important role in anxious states [7, 8]. Furthermore, children with anxiety disorders exhibit more somatic complaints compared to normal control children [9]. RAE children are also known to score higher on the depression/anxiety scale. Thus, anxiety disorders might be related to the pathogenesis of RAE. Control subjects tended to score higher on other externalizing indexes, though these trends were not significant. Internalizing factors such as withdrawn behavior, somatic symptoms, and depression/anxiety refer to a broad class of behaviors in which children direct feelings and emotions inward; these are commonly in contrast to externalizing factors [4]. As shown in Figure 1, although the associations with externalizing factors had no statistical significance, the RAE group scored higher on internalizing indexes and lower on externalizing indexes than the control group. Thus, introspective personality might be a risk factor for refractive accommodative esotropia.

The angle of deviation in RAE is usually variable. To minimize deviation variability, I used the mean of two consecutive deviation measurements for all analyses. The angle of esodeviation correlated with withdrawn behaviors on the CBCL, but the near angle was not associated with the score on any subsection of the CBCL. Withdrawn behavior is defined as the behavioral tendency to isolate oneself from one's peers [10]; nine items on the CBCL indicate withdrawn behavior such as a preference for being alone, sadness, unwillingness to talk, shy demeanor, and withdrawn or underactive behavior. Withdrawn behavior was negatively correlated with the angle of deviation. In other words, RAE children with lower scores on indexes of withdrawn behavior, who are outgoing and active, are more likely to have larger angles of esodeviation. As mentioned earlier, somatic complaints and depression/anxiety might be related to RAE. However, there was no significant correlation between either of these measures and the angle of esodeviation. Therefore, somatic complaints and depression/anxiety might be related to the pathogenesis of RAE. The present study had some limitations. As I mentioned earlier, I did not evaluate related factors such as binocular sensory function or family history. However, these biases can be excluded because the study was designed as a prospective study.

In conclusion, hypermetropic children with high scores on the somatic complaint and depression/anxiety subscales of the CBCL could be at high risk for developing RAE.

## Figures and Tables

**Table 1:** Demographics of RAE patients and controls.

	RAE group (*N* = 22)	Control group (*N* = 34)	*P* value
Gender (M : F)	10 : 12	18 : 16	0.358
Mean age at initial visit (year)	5.0	5.4	0.233
Mean refractive error (diopters)			
Right	4.81 ± 1.94	5.47 ± 1.78	0.262
Left	4.45 ± 2.12	5.22 ± 1.51	
Mean esodeviation (prism diopter)			
Near	30.6 ± 11.0	—	
Far	20.0 ± 12.8		

RAE: refractive accommodative esotropia.

**Table 2:** Correlation between angle of esodeviation and K-CBCL.

	Near angle	Far angle
Withdrawn	−0.248	**−0.653** ^*^
Somatic complaints	−0.365	−0.304
Depression/anxiety	0.134	−0.121
Social problem	0.050	−0.251
Thought problem	0.010	0.198
Attention problem	0.465	−0.411
Delinquent behavior	−0.371	−0.453
Aggressive behavior	−0.028	−0.278

^*^ *P* = 0.042.
